# Randomized Trial of Ceftazidime-Avibactam vs Meropenem for Treatment of Hospital-Acquired and Ventilator-Associated Bacterial Pneumonia (REPROVE): Analyses per US FDA–Specified End Points

**DOI:** 10.1093/ofid/ofz149

**Published:** 2019-04-25

**Authors:** Antoni Torres, Doug Rank, David Melnick, Ludmyla Rekeda, Xiang Chen, Todd Riccobene, Ian A Critchley, Hassan D Lakkis, Dianna Taylor, Angela K Talley

**Affiliations:** 1 Servei de Pneumologia, University of Barcelona, IDIBAPS, CIBERES, ICREA Academia, Hospital Clinic, Barcelona, Spain; 2 Clinical Development, Allergan plc, Madison, New Jersey; 3 Biostatistics, Allergan plc, Madison, New Jersey; 4 Clinical Pharmacology, Allergan plc, Madison, New Jersey; 5 Clinical Microbiology, Allergan plc, Irvine, California; 6 Taylormade Health Ltd, Warrington, UK

**Keywords:** ceftazidime-avibactam, hospital-acquired pneumonia, meropenem, nosocomial pneumonia, ventilator-associated pneumonia

## Abstract

**Background:**

Hospital-acquired and ventilator-associated pneumonia (HAP/VAP; nosocomial pneumonia) due to Gram-negative pathogens are associated with significant morbidity and mortality; treatment options for multidrug-resistant infections are limited. The pivotal phase III REPROVE trial evaluated the efficacy of ceftazidime-avibactam (CAZ-AVI) vs meropenem in the treatment of patients with HAP/VAP. Study results for prespecified analyses per US Food and Drug Administration–recommended trial end points are reported here.

**Methods:**

Hospitalized adults with HAP/VAP proven or suspected to be caused by a Gram-negative pathogen were randomized 1:1 to receive CAZ-AVI or meropenem for 7 to 14 days. The primary outcome was 28-day all-cause mortality in the intent-to-treat (ITT) population. Secondary outcomes included clinical cure at test of cure (TOC) in the ITT and microbiological ITT (micro-ITT) populations, and safety and tolerability throughout the study.

**Results:**

hundred seventy randomized patients received treatment and were included in the ITT population (CAZ-AVI, n = 436; meropenem, n = 434). CAZ-AVI was noninferior to meropenem for the primary end point (28-day all-cause mortality; ITT) based on the prespecified 10% noninferiority margin (CAZ-AVI, 9.6%; meropenem, 8.3%; difference, 1.5%; 95% confidence interval [CI], –2.4% to 5.3%) and for the clinical cure end point in the ITT population based on a prespecified –10% noninferiority margin (CAZ-AVI, 67.2%; meropenem, 69.1%; difference, −1.9%; 95% CI, –8.1% to 4.3%). Clinical cure rates at TOC for patients infected with CAZ-nonsusceptible pathogens were similar (CAZ-AVI, 75.5%; meropenem, 71.2%; micro-ITT). Safety data were consistent with established safety profiles for both agents.

**Conclusions:**

CAZ-AVI provides an important new treatment option for HAP/VAP due to Gram-negative pathogens, including CAZ-nonsusceptible strains.

Hospital-acquired and ventilator-associated bacterial pneumonia (HAP/VAP; nosocomial pneumonia) are collectively the leading cause of death due to hospital-acquired infections worldwide [1–4]. Gram-negative bacilli, including Enterobacteriaceae and *Pseudomonas aeruginosa*, are the most common etiologic HAP/VAP pathogens; treatment is complicated by the increasing prevalence of β-lactamase-producing, multidrug-resistant (MDR) strains [5, 6].

Ceftazidime-avibactam (CAZ-AVI) combines a third-generation cephalosporin with the non-β-lactam β-lactamase inhibitor avibactam, which exhibits broad-spectrum inhibition of clinically relevant serine β-lactamases, including AmpC, extended-spectrum β-lactamases (ESBLs), *Klebsiella pneumoniae* carbapenemase (KPC), and OXA-48 enzymes [7–9]. CAZ–AVI has demonstrated activity against common Gram-negative HAP/VAP pathogens [10–12], including certain drug-resistant (ESBL- and KPC-producing) Enterobacteriaceae and MDR *P. aeruginosa* strains, which are classified as serious and/or urgent threats to US public health [13, 14].

The clinical efficacy of CAZ-AVI for the treatment of serious Gram-negative bacterial infections has been previously established in phase III trials of complicated intra-abdominal and complicated urinary tract infections, which each included subsets of patients with infections due to ceftazidime-nonsusceptible (CAZ-NS) Gram-negative pathogens, supporting approval for both indications in the United States and Europe [10–12].

The global phase III REPROVE study (ClinicalTrials.gov, NCT01808092; EudraCT 2012-004006-96) evaluated the efficacy and safety of CAZ–AVI vs meropenem in the treatment of hospitalized adults with HAP/VAP (nosocomial pneumonia) due to Gram-negative pathogens, including CAZ-NS strains. Primary efficacy end points and analysis populations for the study were separately defined for the US Food and Drug Administration (FDA) and the European Medicines Agency (EMA) to comply with the respective regulatory requirements. Study results presented here for analyses according to the FDA-specified trial end points supported the recent FDA approval of CAZ–AVI for the treatment of adults with HAP/VAP, representing the first Gram-negative antimicrobial approved in the United States for this indication in over 15 years [15]. Results of the study according to the primary end points and analysis populations defined for the EMA have been previously reported [16].

## METHODS

### Study Design

The REPROVE study design (Supplementary Figure 1) and conduct were previously described [16]. Additional details relevant to the summary of data for the US analyses are presented here. The study was conducted in accordance with the Declaration of Helsinki, Good Clinical Practice guidelines, and applicable regulatory requirements. The study protocol and amendments were approved by local ethics committees and/or institutional review boards.

### Study Population

Patients were recruited in 24 countries. Eligible patients included hospitalized adults (aged 18–90 years) with HAP, defined as pneumonia with onset ≥48 hours after admission or within 7 days of discharge from an inpatient care facility, or VAP, defined as a parenchymal lung infection arising ≥48 hours after endotracheal intubation and mechanical ventilation. Key inclusion and exclusion criteria are provided in Supplementary Table 1.

### Study Procedures

Patients were randomized 1:1 to receive either CAZ-AVI 2.5 g (2.0 g ceftazidime + 0.5 g avibactam) every 8 hours (q8h) intravenously (IV) over 2 hours (as approved for the treatment of complicated urinary tract and intra-abdominal infections) plus meropenem placebo, or meropenem 1 g q8h IV over 30 minutes plus CAZ-AVI placebo for 7 to 14 days. Treatment doses for both drugs were adjusted in patients with moderate to severe renal impairment at baseline (MSRIB; creatinine clearance [CrCl] 16–50 mL/min) (Supplementary Table 2). A protocol amendment in year 3 (of 4) of the study increased the CAZ-AVI dose for patients with MSRIB by 50% (MSRIB_new_), consistent with the approved dose recommendations [7] based on emerging data from the phase III CAZ-AVI program [11]. From randomization, patients could receive empiric open-label linezolid or vancomycin to cover for Gram-positive pathogens and/or empiric open-label amikacin or other aminoglycoside for possible MDR Gram-negative organisms for up to 72 hours, pending pathogen identification and susceptibility results, after which open-label therapy was de-escalated as appropriate.

### Outcome Measures

The primary end point for the US analysis was 28-day all-cause mortality (death from any cause by the day 28 visit [28–32 days from randomization]) in the intent-to-treat (ITT) population. Key secondary end points were clinical cure at test of cure (TOC; 21–25 days from randomization) in the ITT population and 28-day all-cause mortality in the microbiological ITT (micro-ITT) population. Additional secondary outcomes included clinical and microbiological responses in the micro-ITT population (including patients infected with CAZ-NS Gram-negative pathogens) and safety/tolerability throughout the study (Supplementary Table 3). Clinical and microbiological response definitions and definitions of the US analysis populations are provided in Supplementary Tables 4 and 5, respectively.

### Statistical Analysis

Noninferiority for the US primary end point was to be concluded if the upper limit of the 2-sided 95% confidence interval (CI) for the difference in 28-day all-cause mortality rate between treatments (CAZ-AVI minus meropenem) was <10%. The sample size ensured ≥90% power for a 10% noninferiority margin. Sensitivity and subgroup analyses for the primary end point were also performed. Noninferiority for the key secondary end point, clinical cure at TOC (ITT population), was to be concluded if the lower limit of the 2-sided 95% CI for the difference between treatments was above –10%. Details on statistical methodology are provided in the Supplementary Data.

## RESULTS

### Patient Characteristics

**Figure 1.**
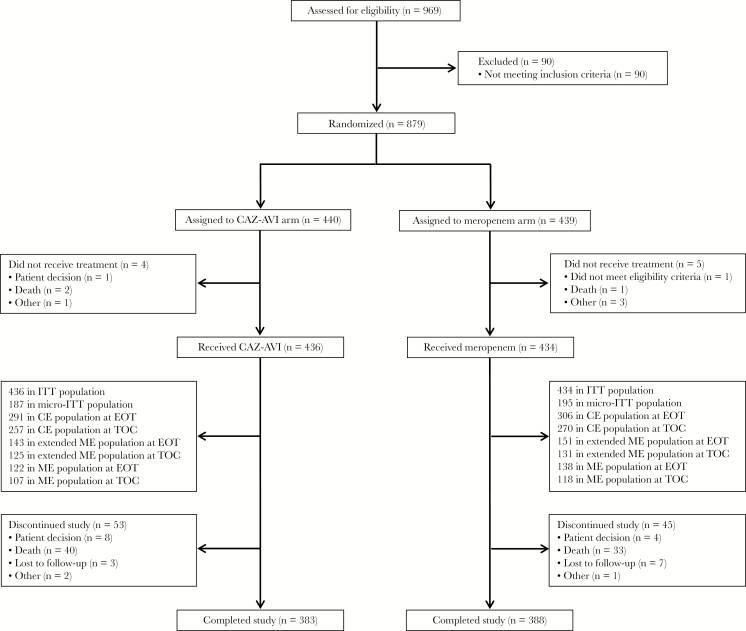
Patient disposition. One patient in the meropenem arm completed the TOC visit (out of window) and the final protocol follow-up visit on the same day and was treated as having neither completed nor discontinued the study. Abbreviations: CAZ-AVI, ceftazidime-avibactam; CE, clinically evaluable; EOT, end of treatment; ITT, intent to treat; ME, microbiologically evaluable; micro-ITT, microbiological ITT; TOC, test of cure.

Between April 2013 and January 2016, 879 patients were randomized; 870 received treatment and were included in the ITT population (CAZ-AVI, n = 436; meropenem, n = 434) (Figure 1). Median exposure to IV study drug in both treatment arms was 10 days. Patient demographics and disease characteristics were well balanced between treatment arms (Table 1) and were as expected for patients with HAP/VAP. Most patients (73.8%) had normal renal function or mild renal impairment (CrCl >50 and ≤150 mL/min) at baseline, and 14.0% had potentially augmented renal clearance (CrCl >150 mL/min). Among 102 (11.7%) patients in the ITT population with MSRIB (CrCl 16–50 mL/min), 62 (31 in each treatment arm) were enrolled before the protocol amendment for the increase in CAZ-AVI dose (MSRIB_orig_), and 40 (CAZ-AVI, n = 21; meropenem, n = 19) were enrolled after the protocol amendment (MSRIB_new_). As allowed per protocol, 81% of patients in both treatment arms received concomitant aminoglycoside antibiotics at any point up to end of treatment (EOT); for most patients, exposure was ≤72 hours.

**Table 1.** **Patient Demographic and Baseline Clinical Characteristics (ITT Population**)

Characteristic	CAZ-AVI (n = 436)	Meropenem (n = 434)	Total (n = 870)
Age, mean (SD), y	62.8 (16.7)	62.8 (17.6)	62.8 (17.2)
<65 y, n (%)	200 (45.9)	201 (46.3)	401 (46.1)
≥75 y, n (%)	129 (29.6)	135 (31.1)	264 (30.3)
Male, n (%)	325 (74.5)	320 (73.7)	645 (74.1)
Region, n (%)			
Western Europe	37 (8.5)	34 (7.8)	71 (8.2)
Eastern Europe	113 (25.9)	109 (25.1)	222 (25.5)
China	143 (32.8)	145 (33.4)	288 (33.1)
Rest of world	143 (32.8)	146 (33.6)	289 (33.2)
Race, n (%)			
White	181 (41.5)	189 (43.5)	370 (42.5)
Asian	245 (56.2)	236 (54.4)	481 (55.3)
Other	10 (2.3)	9 (2.1)	19 (2.2)
BMI, mean (SD), kg/m^2^	23.8 (6.0)	23.6 (5.2)	23.7 (5.6)
APACHE II score, mean (SD)	14.6 (4.1)	15.0 (4.1)	14.8 (4.1)
APACHE II category, n (%)			
<10	3 (0.7)	2 (0.5)	5 (0.6)
10–19	376 (86.2)	369 (85.0)	745 (85.6)
20–30	57 (13.1)	62 (14.3)	119 (13.7)
Renal function category,^a^ n (%)			
Normal renal function/mild renal impairment (CrCl >50–150 mL/min)	324 (74.3)	318 (73.3)	642 (73.8)
Moderate or severe renal impairment (CrCl 16–50 mL/min)	52 (11.9)	50 (11.5)	102 (11.7)
MSRIB_orig_	31 (7.1)	31 (7.1)	62 (7.1)
MSRIB_new_	21 (4.8)	19 (4.4)	40 (4.6)
Augmented (CrCl >150 mL/min)	58 (13.3)	64 (14.7)	122 (14.0)
HAP/VAP subtype, n (%)			
HAP	291 (66.7)	289 (66.6)	580 (66.7)
VAP	145 (33.3)	145 (33.4)	290 (33.3)
Type of infection (VAP patients), n (%)			
Early VAP	36 (8.3)	54 (12.4)	90 (10.3)
Late VAP	109 (25.0)	91 (21.0)	200 (23.0)
Mechanically ventilated at baseline, n (%)	193 (44.3)	186 (42.9)	379 (43.6)
VAP	145 (33.3)	145 (33.4)	290 (33.3)
Ventilated HAP	48 (11.0)	41 (9.4)	89 (10.2)
Bacteremic, n (%)	24 (5.5)	18 (4.1)	42 (4.8)
Micro-ITT population	21/187 (11.2)	15/195 (7.7)	36/382 (9.4)
Infection type, n (%)			
Monomicrobial infection	141 (32.3)	130 (30.0)	271 (31.1)
Micro-ITT population	111/187 (59.4)	107/195 (54.9)	218/382 (57.1)
Polymicrobial infection	76 (17.4)	89 (20.5)	165 (19.0)
Micro-ITT population	76/187 (40.6)	88/195 (45.1)	164/382 (42.9)
No study-qualifying pathogen identified	219 (50.2)	215 (49.5)	434 (49.9)
Prior systemic Gram-negative antibiotic exposure,^b^ n (%)			
0–24 h	322 (73.9)	323 (74.4)	645 (74.1)
>24 h	114 (26.1)	111 (25.6)	225 (25.9)
Concomitant aminoglycoside use up to EOT,^c^ n (%)			
0–72 h	334 (76.6)	344 (79.3)	678 (77.9)
>72 h	102 (23.4)	90 (20.7)	192 (22.1)

Abbreviations: APACHE, Acute Physiology and Chronic Health Evaluation; BMI, body mass index; CAZ-AVI, ceftazidime-avibactam; CrCl, creatinine clearance; EOT, end of treatment; HAP, hospital-acquired bacterial pneumonia; ITT, intent to treat; micro-ITT, microbiological ITT; MSRIB, moderate to severe renal impairment at baseline; MSRIB_new_, MSRIB cohort enrolled after protocol amendment for increased CAZ-AVI dose; MSRIB_orig_, MSRIB cohort enrolled before protocol amendment for increased CAZ-AVI dose; VAP, ventilator-associated bacterial pneumonia.

^a^Based on estimated CrCl per the Cockcroft-Gault method and local laboratory data.

^b^In the 72 hours before randomization.

^c^Exploratory analysis based on blinded review of postbaseline data.

The micro-ITT population comprised 382 patients (CAZ-AVI, n = 187; meropenem, n = 195) for whom ≥1 Gram-negative pathogen was isolated from baseline respiratory and/or blood culture, including 164 (43%) patients with polymicrobial infections, of whom 73 (19%) had both Gram-positive and Gram-negative aerobic pathogens isolated.

As expected, most infections were due to Enterobacteriaceae (CAZ-AVI, 133/187 [71.1%]; meropenem, 147/195 [75.4%]) (Supplementary Table 6). The distribution of baseline pathogens was similar between treatment arms, with the exception of *P. aeruginosa*, which was more prevalent in the CAZ-AVI arm. Among patients in the micro-ITT population, 108 (28.3%) were infected with ≥1 Gram-negative pathogen that was nonsusceptible to ceftazidime based on Clinical and Laboratory Standards Institute (CLSI)–defined criteria [17] for ceftazidime-resistant and -intermediate susceptibility categories (ie, minimum inhibitory concentration [MIC] ≥8 mg/L for Enterobacteriaceae and ≥16 mg/L for *P. aeruginosa*), including 53 patients with *K. pneumoniae* and 28 with *P. aeruginosa*. In a subset of Gram-negative pathogens that met phenotypic (MIC) screening criteria for the presence of a β-lactamase, genotypic testing identified certain ESBL groups (eg, TEM-1, SHV-12, CTX-M-15, and KPC-2 carbapenemase) and AmpC that were expected to be inhibited by avibactam in isolates from 115/382 (30.1%) patients in the micro-ITT population.

The MICs required to inhibit ≥90% of isolates with CAZ-AVI or ceftazidime for 339 Enterobacteriaceae isolates were 0.5 and 64 µg/mL, respectively, and 8 and 64 µg/mL for 111 *P. aeruginosa* isolates (micro-ITT population). Thus, the CAZ-AVI MIC distributions for Enterobacteriaceae and *P. aeruginosa* isolates were left-shifted compared with those for ceftazidime alone (Supplementary Figure 2). Two *K. pneumoniae* isolates and 9 *P. aeruginosa* isolates were nonsusceptible to CAZ-AVI, and 6 Enterobacteriaceae isolates and 34 *P. aeruginosa* isolates were nonsusceptible to meropenem based on CLSI criteria.

### Efficacy Results

#### Twenty-Eight-Day All-Cause Mortality

CAZ-AVI was noninferior to meropenem with respect to the US primary end point (28-day all-cause mortality; ITT population) based on a 10% noninferiority margin (CAZ-AVI, 42/436 [9.6%]; meropenem, 36/434 [8.3%]; difference, 1.5% [Kaplan-Meier estimate]; 95% CI, −2.4 to 5.3). Results for the micro-ITT population were consistent with the primary analysis (Table 2). Likewise, mortality rates among the subset of patients with CAZ-NS pathogens were similar between treatment arms (CAZ-AVI, 8.2% [4/49]; meropenem, 8.5% [5/59]).

**Table 2.** Twenty-Eight-Day All-Cause Mortality (ITT and Micro-ITT Populations): Kaplan-Meier Estimates and Noninferiority Hypothesis Test

Analysis Population	Patient Deaths		Between-Arm Difference^a^ (95% CI), %
	CAZ-AVI, n/N (%) [KM%]	Meropenem, n/N (%) [KM%]	
ITT	42/436 (9.6) [9.9]	36/434 (8.3) [8.4]	1.5 (−2.4 to 5.3)
Micro-ITT	22/187 (11.8)	19/195 (9.7)	2.1 (–4.1 to 8.4)
CAZ-NS	4/49 (8.2)	5/59 (8.5)	−0.1

Abbreviations: CAZ-AVI, ceftazidime-avibactam; CAZ-NS, ceftazidime-nonsusceptible; CI, confidence interval; ITT, intent to treat; KM, Kaplan-Meier; micro-ITT, microbiological ITT.

^a^Differences based on the KM estimates of the cumulative survival proportions for each treatment arm up to day 28; CIs for the difference were calculated based on Greenwood’s variance estimates.

**Figure 2.**
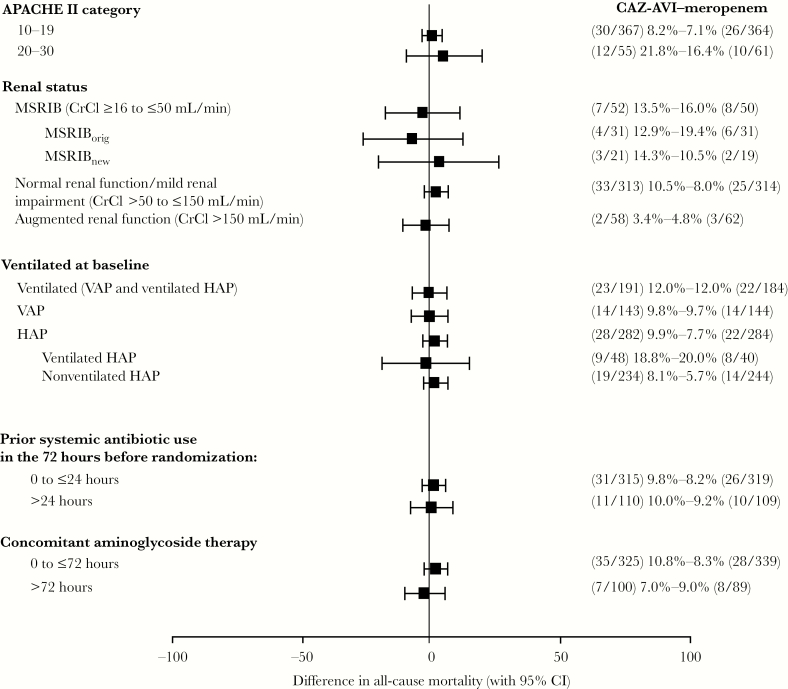
Subgroup analyses: 28-day all-cause mortality by patient baseline characteristics (ITT population). Based on difference between treatment arms in proportions of patients who died up to day 28; CIs for the difference were calculated using the unstratified Miettinen and Nurminen method. Abbreviations: APACHE, Acute Physiology and Chronic Health Evaluation; CAZ-AVI, ceftazidime-avibactam; CI, confidence interval; CrCl, creatinine clearance; HAP, hospital-acquired bacterial pneumonia; ITT, intent to treat; MSRIB, moderate to severe renal impairment at baseline; MSRIB_new_, MSRIB cohort enrolled after protocol amendment for increased CAZ-AVI dose; MSRIB_orig_, MSRIB cohort enrolled before protocol amendment for increased CAZ-AVI dose; VAP, ventilator-associated bacterial pneumonia.

Sensitivity analyses of the US primary end point (Supplementary Data) using multiple imputation of missing data at the day 28 visit, or excluding data for patients who were lost to follow-up, were consistent with the primary analysis (Supplementary Table 7), as were subgroup analyses based on key patient characteristics for which mortality rates were broadly similar between the CAZ-AVI and meropenem arms (Figure 2). All CIs for the difference between treatment arms included 0.

#### Clinical Cure

**Figure 3.**
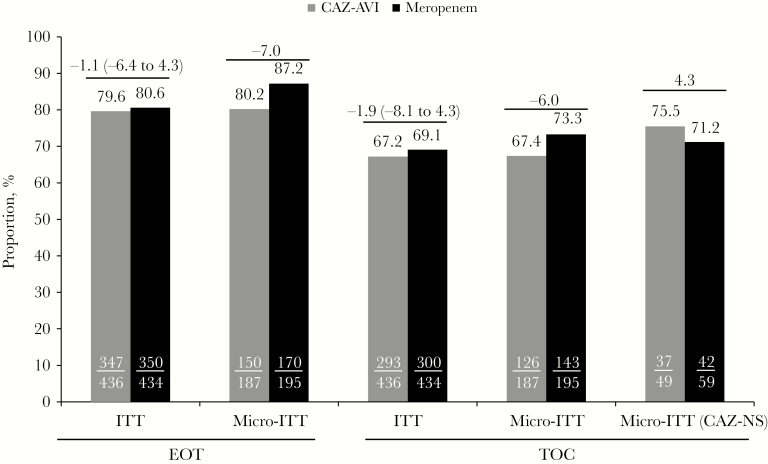
Clinical cure rates at EOT and TOC visits (ITT and micro-ITT populations). CIs for the difference between treatment arms were calculated using the unstratified Miettinen and Nurminen method. CI was not calculated for micro-ITT population because of small patient numbers. Abbreviations: CAZ-AVI, ceftazidime-avibactam; CAZ-NS, ceftazidime-nonsusceptible; CI, confidence interval; EOT, end of treatment; ITT, intent to treat; micro-ITT, microbiological ITT; TOC, test of cure.

CAZ-AVI was noninferior to meropenem with respect to the key secondary end point (clinical cure at TOC; ITT population) based on a –10% noninferiority margin; clinical cure was achieved in 293 (67.2%) patients in the CAZ-AVI arm and 300 (69.1%) patients in the meropenem arm (difference, –1.9; 95% CI, –8.1 to 4.3). In the micro-ITT population, including patients infected with CAZ-NS pathogens, 75.5% and 71.2% in the CAZ-AVI and meropenem arms, respectively, achieved clinical cure at TOC (Figure 3). Clinical cure rates in both populations were slightly higher at EOT and were comparable between treatment arms. Favorable clinical response rates at TOC by baseline pathogen were generally similar between treatment arms across all baseline pathogens, although definitive comparisons are limited in these individual pathogen subsets (Table 3).

**Table 3.** Favorable Clinical and Microbiological Response Rates at TOC by Baseline Pathogen^a^ (Micro-ITT Population)

Pathogen Group/Pathogen	Per-Patient Clinical Cure^b^		Per-Pathogen Microbiological Eradication^c^	
	CAZ-AVI, n/N (%)	Meropenem, n/N (%)	CAZ-AVI, n/N (%)	Meropenem, n/N (%)
Aerobic Gram-negative	126/187 (67.4)	143/195 (73.3)	155/256 (60.5)	174/267 (65.2)
Enterobacteriaceae	92/133 (69.2)	108/147 (73.5)	111/168 (66.1)	126/182 (69.2)
*Enterobacter aerogenes*	5/8 (62.5)	4/9 (44.4)	5/8 (62.5)	6/9 (66.7)
*Enterobacter cloacae*	25/29 (86.2)	13/23 (56.5)	22/29 (75.9)	14/23 (60.9)
*Escherichia coli*	12/22 (54.5)	17/23 (73.9)	14/22 (63.6)	16/23 (69.6)
*Klebsiella pneumoniae*	44/65 (67.7)	56/75 (74.7)	39/65 (60.0)	54/75 (72.0)
*Proteus mirabilis*	12/14 (85.7)	9/12 (75.0)	11/14 (78.6)	8/12 (66.7)
*Serratia marcescens*	11/15 (73.3)	12/13 (92.3)	10/15 (66.7)	8/13 (61.5)
Gram-negative pathogens other than Enterobacteriaceae	54/85 (63.5)	61/84 (72.6)	44/88 (50.0)	48/85 (56.5)
*Haemophilus influenzae*	13/16 (81.3)	20/25 (80.0)	14/16 (87.5)	23/25 (92.0)
*Pseudomonas aeruginosa*	38/64 (59.4)	37/51 (72.5)	24/64 (37.5)	20/51 (39.2)
CAZ-NS pathogens^d^	37/49 (75.5)	42/59 (71.2)	35/52 (67.3)	33/64 (51.6)
Enterobacteriaceae	29/36 (80.6)	31/45 (68.9)	31/40 (77.5)	29/47 (61.7)
*E. aerogenes*	3/4 (75.0)	2/2 (100.0)	3/4 (75.0)	2/2 (100.0)
*E. cloacae*	6/6 (100.0)	4/6 (66.7)	5/6 (83.3)	5/6 (83.3)
*E. coli*	4/6 (66.7)	5/8 (62.5)	4/6 (66.7)	4/8 (50.0)
*K. pneumoniae*	17/22 (77.3)	22/31 (71.0)	17/22 (77.3)	18/31 (58.1)
*P. aeruginosa*	7/12 (58.3)	13/16 (81.3)	4/12 (33.3)	4/16 (25.0)

Abbreviations: CAZ-AVI, ceftazidime-avibactam; CAZ-NS, ceftazidime-nonsusceptible; CLSI, Clinical and Laboratory Standards Institute; micro-ITT, microbiological intent to treat; TOC, test of cure.

^a^Respiratory tract or blood source. Only pathogens with a combined total of ≥10 isolates across treatment arms (≥5 for CAZ-NS subset) are presented. Multiple isolates of the same species from the same patient are counted only once regardless of source (respiratory tract or blood) using the isolate with the highest minimum inhibitory concentration to study drug received.

^b^Proportion of patients assessed as a clinical cure at TOC visit; percentages are based on the total number of patients in the subgroup (N).

^c^Defined as eradication or presumed eradication of the baseline pathogen at the TOC visit; percentages are based on the total number of unique pathogens (N).

^d^CAZ-NS designation was determined according to CLSI criteria for the ceftazidime-resistant and -intermediate categories [17].

#### Microbiological Response

**Figure 4.**
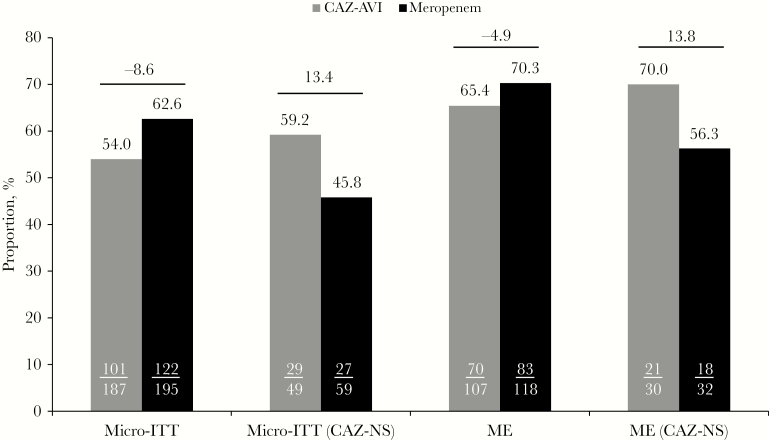
Per-patient favorable microbiological response at TOC (micro-ITT and ME populations). Abbreviations: CAZ-AVI, ceftazidime-avibactam; CAZ-NS, ceftazidime-nonsusceptible; ME, microbiologically evaluable; micro-ITT, microbiological intent to treat; TOC, test of cure.

The overall per-patient favorable microbiological response (eradication or presumed eradication) rates at TOC were similar between treatment arms in the micro-ITT and microbiologically evaluable populations, including patients infected with CAZ-NS pathogens (Figure 4). Per-pathogen favorable microbiological response rates at TOC in the micro-ITT population were similar between treatment arms across all baseline pathogens, including CAZ-NS strains (Table 3). Overall eradication rates in the micro-ITT population for CAZ-NS Gram-negative pathogens were 67.3% (35/52) in the CAZ-AVI arm and 51.6% (33/64) in the meropenem arm.

Among the subset of pathogens with an unfavorable microbiological response due to persistence of the baseline isolate, an increase in postbaseline MIC for study drug received (≥4-fold) was observed for 4/79 (5.1%) and 14/75 (18.7%) pathogens in the CAZ-AVI and meropenem arms, respectively, including 2/65 (3.1%) and 3/75 (4.0%) *K. pneumoniae* isolates in the CAZ-AVI and meropenem arms, respectively. Among *P. aeruginosa* isolates, however, 11/51 (21.6%) in the meropenem arm had an increase in postbaseline MIC, compared with 2/64 (3.1%) in the CAZ-AVI arm. The per-pathogen microbiological responses by MIC to study drug received were assessed for Enterobacteriaceae and *P. aeruginosa.* No trend in unfavorable microbiological outcomes was observed over the MIC range among Enterobacteriaceae isolates (CAZ-AVI MIC, 0.015 to 4 µg/mL; meropenem MIC, 0.008 to >8 µg/mL), nor among clinical isolates of *P. aeruginosa* (CAZ-AVI MIC, 0.5 to >256 µg/mL; meropenem MIC, 0.06 to >8 µg/mL), suggesting that exposure to the study drug for both CAZ-AVI- and meropenem-treated patients was adequate.

### Safety

Overall, 74% of patients in both treatment arms experienced ≥1 adverse event (AE) during the study. The majority of reported AEs were of mild or moderate intensity and were balanced between treatment arms (Table 4). The overall type and distribution of AEs were consistent with what is expected for patients with HAP/VAP and/or the known safety profile for both drugs. AEs resulted in discontinuation of study drug in 16 (3.7%) patients in the CAZ-AVI arm and 13 (3.0%) patients in the meropenem arm.

**Table 4.** Adverse Events Up to Final Patient Follow-up Visit (Safety Population)

Summary^a^	CAZ-AVI (n = 436), n (%)	Meropenem (n = 434), n (%)
Any AE	323 (74.1)	321 (74.0)
Any AE with outcome of death^b^	26 (6.0)	23 (5.3)
Any SAE	79 (18.1)	59 (13.6)
Any AE leading to discontinuation of study drug	16 (3.7)	13 (3.0)
Any AE of severe intensity	68 (15.6)	55 (12.7)
AEs reported in ≥2% of patients in either treatment arm by SOC/ preferred term		
Infections and infestations		
Urinary tract infection	11 (2.5)	15 (3.5)
Pneumonia	10 (2.3)	12 (2.8)
Blood and lymphatic system disorders		
Anemia	25 (5.7)	19 (4.4)
Metabolism and nutrition disorders		
Hypokalemia	47 (10.8)	37 (8.5)
Hyponatremia	10 (2.3)	7 (1.6)
Psychiatric disorders		
Insomnia	6 (1.4)	11 (2.5)
Cardiac disorders		
Cardiac failure	9 (2.1)	6 (1.4)
Atrial fibrillation	6 (1.4)	9 (2.1)
Vascular disorders		
Hypertension	14 (3.2)	17 (3.9)
Hypotension	10 (2.3)	9 (2.1)
Respiratory, thoracic, and mediastinal disorders		
Pleural effusions	10 (2.3)	9 (2.1)
Respiratory failure	10 (2.3)	6 (1.4)
Gastrointestinal disorders		
Diarrhea	67 (15.4)	67 (15.4)
Constipation	25 (5.7)	32 (7.4)
Vomiting	25 (5.7)	24 (5.5)
Nausea	14 (3.2)	7 (1.6)
Skin and subcutaneous tissue disorders		
Decubitus ulcer	10 (2.3)	6 (1.4)
Rash	9 (2.1)	16 (3.7)
General disorders and administration site conditions		
Peripheral edema	17 (3.9)	16 (3.7)
Pyrexia	14 (3.2)	16 (3.7)
Investigations		
Increased alanine aminotransferase	17 (3.9)	19 (4.4)
Increased aspartate aminotransferase	17 (3.9)	17 (3.9)

Abbreviations: AE, adverse event; CAZ-AVI, ceftazidime-avibactam; SAE, serious AE; SOC, system organ class.

^a^Patients with multiple AEs were counted only once for each specific AE category, SOC, and/or preferred term.

^b^Deaths due to disease progression were not included in this category.

Serious AEs (SAEs) were reported for 79 (18.1%) patients in the CAZ-AVI arm and 59 (13.6%) patients in the meropenem arm (Table 4). Consistent with the disease under study, the most common SAEs (reported in ≥1% of patients in either treatment arm) were pneumonia, respiratory failure, and sepsis. SAEs assessed by the investigator as possibly related to study drug were reported for 5 (1.1%) CAZ-AVI and 2 (0.5%) meropenem patients and were generally consistent with the established safety profile of the study drugs [16].

## Discussion

In the setting of a complex and evolving regulatory environment and the unique challenges to conducting randomized comparative studies in patients with HAP/VAP, there have been few registrational HAP/VAP trials conducted and no new antimicrobials approved for this indication by the FDA over the last decade [18–20]. Based on the positive results from the REPROVE study presented here, in 2018 the FDA approved CAZ-AVI for treatment of patients with HAP/VAP. CAZ-AVI is thus the first new Gram-negative antibiotic approved in the United States for this indication in over 15 years [18–21].

CAZ-AVI was noninferior to meropenem with respect to the FDA-specified primary end point of 28-day all-cause mortality in the ITT population and with respect to the key secondary end point of clinical cure at TOC in the ITT population. Results for the micro-ITT population and key patient subgroups were consistent with these analyses. Per-patient and per-pathogen favorable clinical and microbiological response rates were generally high and similar between treatment arms across all visits and analysis populations, including the subset of patients with CAZ-NS pathogens, which represented 28% of the micro-ITT population. Among patients infected with *P. aeruginosa*, a substantially higher proportion in the meropenem arm had persistence of the baseline pathogen associated with a ≥4-fold increase in postbaseline MIC to study drug received, raising concerns for potential treatment-emergent resistance. This is consistent with prior observations for imipenem-cilastatin in randomized controlled nosocomial pneumonia trials using other β-lactam or fluoroquinolone comparators, in which a higher incidence of treatment-emergent *P. aeruginosa* resistance was reported among carbapenem-treated patients [22–24].

CAZ-AVI was well tolerated in patients with HAP/VAP; safety observations were consistent with the established safety profile for CAZ-AVI and/or ceftazidime [10–12, 25]. Although numerically higher among CAZ-AVI-treated patients, reported SAEs were consistent with the underlying illness and/or anticipated clinical course and do not appear to represent a safety signal for CAZ-AVI in this population.

Taken together, data from REPROVE indicate that CAZ-AVI is an effective therapy for patients with HAP/VAP due to Gram-negative bacteria, including CAZ-NS strains. Study results demonstrating noninferiority vs a carbapenem comparator were robust across multiple sensitivity and subgroup analyses for the US primary end point and are consistent with previously reported analyses demonstrating noninferiority according to EMA-specified end points for HAP/VAP trials, reinforcing the robust nature of the data [16].

Of note, the study provides the first phase III assessment of the currently labeled CAZ-AVI dose regimens for patients with CrCl of 16–50 mL/min [7]. The original protocol-defined CAZ-AVI renal dosage adjustments (MSRIB_orig_) in REPROVE were consistent with those utilized in the earlier phase III CAZ-AVI studies [10–12] but were modified during the trial based on emerging data from the phase III cIAI study (RECLAIM), which indicated a potential for underdosing in MSRIB patients with rapidly improving renal function early in the course of treatment [11]. Based on updated population pharmacokinetic analyses and pharmacokinetic/pharmacodynamic target attainment simulations, the dosage regimens were changed for patients with MSRIB such that the total daily CAZ-AVI dose was increased by 50% (MSRIB_new_) in order to ensure that CAZ-AVI exposure in patients with fluctuating renal function would be maintained within the range observed for patients with CrCl >50 mL/min. Extensive prespecified comparative analyses for the original (MSRIB_orig_) and revised (MSRIB_new_) dose regimens in this study provide reassuring data that the labeled dose recommendations are safe and effective in patients with renal impairment [26].

Receipt of prior and/or concomitant antibiotics did not appear to confound the assessment of efficacy; subgroup and additional exploratory analyses evaluating the impact of potentially effective prior or concomitant antibiotics demonstrated minimal variation in mortality rates across subgroups of patients who received the FDA guidance–recommended exposures of ≤24 hours of prior and/or ≤72 hours of concomitant Gram-negative antibacterial therapy, including aminoglycosides, vs those who received >24 hours of prior and/or >72 hours of concomitant Gram-negative therapy [7, 15].

The mortality rates observed in REPROVE were within the range reported for other contemporary HAP and VAP clinical trials [4, 27–30]. A comprehensive review of data from 12 (2008–2013) randomized trials in patients with HAP and/or VAP from the Foundation for the National Institutes of Health (FNIH) Biomarkers Consortium reported mortality rates from 10% to 30% across studies and HAP/VAP subtypes [31], whereas a systematic review of 12 randomized controlled trials, including 3571 patients with VAP, reported 28-day all-cause mortality of 8% to 35% across treatment arms and an overall risk of 19% to 20% [30]. The randomized controlled trials summarized in the current US treatment guidelines noted mortality rates of 8% to 40% across studies and treatment arms [4]. Across several of these HAP/VAP studies, mortality rates for patients treated with a carbapenem-based regimen, as per the comparator arm in the REPROVE study, were frequently among the lowest (<15%).

As noted in other studies, the mortality rates observed in clinical practice may fall near the higher end of the ranges noted above, as inclusion criteria in the controlled clinical trial setting generally restrict enrollment to exclude patients with immunosuppression, hemodialysis, or other comorbidities. However, review of the baseline patient characteristics typically associated with disease severity (eg, older age, APACHE II scores, resistant pathogens) indicates that the REPROVE study enrolled a representative HAP/VAP patient population consistent with that of previous trials [7, 31].

In general, reported mortality rates in patients with nonventilated HAP are lower than in patients with VAP, and the overall mortality rates in REPROVE reflect this. However, patients with ventilated HAP have mortality rates approaching or exceeding those of patients with VAP, denoting this as a distinctly high-risk group [31]. Consistent with this observation, mortality rates in the REPROVE study were highest in patients with ventilated HAP and were balanced across treatment arms (CAZ-AVI, 18.8% [9/48]; meropenem, 20.0% [8/40]). Data for this subgroup are consistent with the FNIH summary of contemporary data [31] and with the primary analysis for the study, reinforcing the conclusion of noninferiority between CAZ-AVI and meropenem in the current trial.

A potential limitation of this study was that meropenem was administered as a 30-minute infusion, consistent with the approved product prescribing information [32], whereas in clinical practice, there is likely some variation in treatment protocols, in which longer infusions are sometimes used for treatment of serious bacterial infections [33–35]. Recruitment of patients in the REPROVE study targeted enrollment from countries with high rates of HAP/VAP due to resistant pathogens. Because the study was originally designed to support approval outside the United States, no US centers participated in the study. However, patients in the study were demographically, clinically, and microbiologically representative of a US HAP/VAP population [7, 27, 31].

In summary, analyses of data from the pivotal REPROVE study demonstrated the noninferiority of CAZ-AVI to meropenem in the treatment of HAP/VAP according to FDA guidance–specified end points. CAZ-AVI therefore offers an important new treatment option and alternative to carbapenems in patients with HAP/VAP caused by Gram-negative pathogens, particularly in the setting of proven or suspected bacterial resistance.

## Supplementary Data

Supplementary materials are available at *Open Forum Infectious Diseases* online. Consisting of data provided by the authors to benefit the reader, the posted materials are not copyedited and are the sole responsibility of the authors, so questions or comments should be addressed to the corresponding author.

Supplementary-Materials

## References

[1] Magill SS, Edwards JR, Bamberg W, et al; Emerging Infections Program Healthcare-Associated Infections and Antimicrobial Use Prevalence Survey Team Multistate point-prevalence survey of health care-associated infections. N Engl J Med 2014; 370:1198–208. 24670166 10.1056/NEJMoa1306801 PMC4648343

[2] Micek ST, Chew B, Hampton N, Kollef MH A case-control study assessing the impact of nonventilated hospital-acquired pneumonia on patient outcomes. Chest 2016; 150:1008–14. 27102181 10.1016/j.chest.2016.04.009 PMC7094544

[3] Melsen WG, Rovers MM, Koeman M, Bonten MJ Estimating the attributable mortality of ventilator-associated pneumonia from randomized prevention studies. Crit Care Med 2011; 39:2736–42. 21765351 10.1097/CCM.0b013e3182281f33

[4] Kalil AC, Metersky ML, Klompas M, et al. Management of adults with hospital-acquired and ventilator-associated pneumonia: 2016 clinical practice guidelines by the Infectious Diseases Society of America and the American Thoracic Society. Clin Infect Dis 2016; 63:e61–e111. 27418577 10.1093/cid/ciw353 PMC4981759

[5] Jones RN Microbial etiologies of hospital-acquired bacterial pneumonia and ventilator-associated bacterial pneumonia. Clin Infect Dis 2010; 51(Suppl 1):S81–7. 20597676 10.1086/653053

[6] Sader HS, Farrell DJ, Flamm RK, Jones RN Antimicrobial susceptibility of gram-negative organisms isolated from patients hospitalised with pneumonia in US and European hospitals: results from the SENTRY Antimicrobial Surveillance Program, 2009–2012. Int J Antimicrob Agents 2014; 43:328–34. 24630306 10.1016/j.ijantimicag.2014.01.007

[7] AVYCAZ (ceftazidime-avibactam). Full Prescribing Information. Dublin, Ireland: Allergan plc; 2018.

[8] Sader HS, Castanheira M, Flamm RK Antimicrobial activity of ceftazidime-avibactam against gram-negative bacteria isolated from patients hospitalized with pneumonia in U.S. medical centers, 2011 to 2015. Antimicrob Agents Chemother 2017; 61:1–10. 10.1128/AAC.02083-16 PMC5365649 28069649

[9] Zhanel GG, Lawson CD, Adam H, et al. Ceftazidime-avibactam: a novel cephalosporin/β-lactamase inhibitor combination. Drugs 2013; 73:159–77. 23371303 10.1007/s40265-013-0013-7

[10] Carmeli Y, Armstrong J, Laud PJ, et al. Ceftazidime-avibactam or best available therapy in patients with ceftazidime-resistant Enterobacteriaceae and *Pseudomonas aeruginosa* complicated urinary tract infections or complicated intra-abdominal infections (REPRISE): a randomised, pathogen-directed, phase 3 study. Lancet Infect Dis 2016; 16:661–73. 27107460 10.1016/S1473-3099(16)30004-4

[11] Mazuski JE, Gasink LB, Armstrong J, et al. Efficacy and safety of ceftazidime-avibactam plus metronidazole versus meropenem in the treatment of complicated intra-abdominal infection: results from a randomized, controlled, double-blind, phase 3 program. Clin Infect Dis 2016; 62:1380–9. 26962078 10.1093/cid/ciw133 PMC4872289

[12] Wagenlehner FM, Sobel JD, Newell P, et al. Ceftazidime-avibactam versus doripenem for the treatment of complicated urinary tract infections, including acute pyelonephritis: RECAPTURE, a phase 3 randomized trial program. Clin Infect Dis 2016; 63:754–62. 27313268 10.1093/cid/ciw378 PMC4996135

[13] Jacob JT, Klein E, Laxminarayan R, et al Vital signs: carbapenem-resistant Enterobacteriaceae. Morb Mortal Wkly Rep 2013; 62:165–70. PMC4604788 23466435

[14] van Duin D, Kaye KS, Neuner EA, Bonomo RA Carbapenem-resistant Enterobacteriaceae: a review of treatment and outcomes. Diagn Microbiol Infect Dis 2013; 75:115–20. 23290507 10.1016/j.diagmicrobio.2012.11.009 PMC3947910

[15] US Department of Health and Human Services, US Food and Drug Administration, Center for Drug Evaluation and Research (CDER). Guidance for industry. Hospital-acquired bacterial pneumonia and ventilator-associated bacterial pneumonia: developing drugs for treatment https://www.fda.gov/downloads/drugs/guidances/ucm234907.pdf. Accessed 29 November 2018.

[16] Torres A, Zhong N, Pachl J, et al. Ceftazidime-avibactam versus meropenem in nosocomial pneumonia, including ventilator-associated pneumonia (REPROVE): a randomised, double-blind, phase 3 non-inferiority trial. Lancet Infect Dis 2018; 18:285–95. 29254862 10.1016/S1473-3099(17)30747-8

[17] Clinical and Laboratory Standards Institute. Performance Standards for Antimicrobial Susceptibility Testing: Twenty-seventh Informational Supplement. CLSI Document M100-S27. Wayne, PA: Clinical and Laboratory Standards Institute; 2017.

[18] Weiss E, Essaied W, Adrie C, et al. Treatment of severe hospital-acquired and ventilator-associated pneumonia: a systematic review of inclusion and judgment criteria used in randomized controlled trials. Crit Care 2017; 21:162. 28655326 10.1186/s13054-017-1755-5 PMC5488424

[19] Knirsch C, Alemayehu D, Botgros R, et al. Improving conduct and feasibility of clinical trials to evaluate antibacterial drugs to treat hospital-acquired bacterial pneumonia and ventilator-associated bacterial pneumonia: recommendations of the Clinical Trials Transformation Initiative Antibacterial Drug Development Project Team. Clin Infect Dis 2016; 63(Suppl 2):S29–36. 27481950 10.1093/cid/ciw258

[20] Stergiopoulos S, Getz KA, Blazynski C Evaluating the completeness of ClinicalTrials.gov. Ther Innov Regul Sci. doi:10.1177/2168479018782885. [Epub ahead of print]. 10.1177/2168479018782885 30048602

[21] Stone GG, Bradford PA, Newell P, Wardman A In vitro activity of ceftazidime-avibactam against isolates in a phase 3 open-label clinical trial for complicated intra-abdominal and urinary tract infections caused by ceftazidime-nonsusceptible Gram-negative pathogens. Antimicrob Agents Chemother. 2017; 61:e01820–16. 27872067 10.1128/AAC.01820-16 PMC5278708

[22] Siempos II, Vardakas KZ, Manta KG, Falagas ME Carbapenems for the treatment of immunocompetent adult patients with nosocomial pneumonia. Eur Respir J 2007; 29:548–60. 17329491 10.1183/09031936.00080206

[23] Joshi M, Metzler M, McCarthy M, et al. Comparison of piperacillin/tazobactam and imipenem/cilastatin, both in combination with tobramycin, administered every 6 h for treatment of nosocomial pneumonia. Respir Med 2006; 100:1554–65. 16487695 10.1016/j.rmed.2006.01.004

[24] Jaccard C, Troillet N, Harbarth S, et al. Prospective randomized comparison of imipenem-cilastatin and piperacillin-tazobactam in nosocomial pneumonia or peritonitis. Antimicrob Agents Chemother 1998; 42:2966–72. 9797234 10.1128/aac.42.11.2966 PMC105974

[25] FORTAZ (ceftazidime). Full Prescribing Information. Research Triangle Park, NC: GlaxoSmithKline; 2007.

[26] Talley AK, Riccobene T, Critchley IA, et al Outcomes among patients with hospital-acquired bacterial pneumonia or ventilator-associated bacterial pneumonia stratified by renal function: subgroup analysis from a phase 3 study of ceftazidime-avibactam. Paper presented at: ASM Microbe; June 7–11, 2018; Atlanta, GA.

[27] Pugh R, Grant C, Cooke RP, Dempsey G Short-course versus prolonged-course antibiotic therapy for hospital-acquired pneumonia in critically ill adults. Cochrane Database Syst Rev 2015; (10): CD007577. 26301604 10.1002/14651858.CD007577.pub3 PMC7025798

[28] Dimopoulos G, Poulakou G, Pneumatikos IA, et al. Short- vs long-duration antibiotic regimens for ventilator-associated pneumonia: a systematic review and meta-analysis. Chest 2013; 144:1759–67. 23788274 10.1378/chest.13-0076

[29] Timsit JF, Zahar JR, Chevret S Attributable mortality of ventilator-associated pneumonia. Curr Opin Crit Care 2011; 17:464–71. 21844801 10.1097/MCC.0b013e32834a5ae9

[30] Arthur LE, Kizor RS, Selim AG, et al Antibiotics for ventilator-associated pneumonia. Cochrane Database Syst Rev 2016; ( 10):CD004267. 27763732 10.1002/14651858.CD004267.pub4 PMC6461148

[31] Foundation for the National Institutes of Health (FNIH) Biomarkers Consortium HABP/VABP Project Team. Considerations for clinical trial design for the study of hospital-acquired bacterial pneumonia and ventilator-associated bacterial pneumonia https://www.regulations.gov/document?D=FDA-2010-D-0589-0027. Accessed 29 November 2018.

[32] Merrem IV (meropenem for injection). Full Prescribing Information. Wilmington, DE: AstraZeneca; 2016.

[33] Capitano B, Nicolau DP, Potoski BA, et al. Meropenem administered as a prolonged infusion to treat serious Gram-negative central nervous system infections. Pharmacotherapy 2004; 24:803–7. 15222672 10.1592/phco.24.8.803.36070

[34] Lorente L, Lorenzo L, Martín MM, et al. Meropenem by continuous versus intermittent infusion in ventilator-associated pneumonia due to Gram-negative bacilli. Ann Pharmacother 2006; 40:219–23. 16449546 10.1345/aph.1G467

[35] Wang D Experience with extended-infusion meropenem in the management of ventilator-associated pneumonia due to multidrug-resistant *Acinetobacter baumannii*. Int J Antimicrob Agents 2009; 33:290–1. 19091523 10.1016/j.ijantimicag.2008.09.012

