# Supplementary material for: Randomized Trial of Ceftazidime-Avibactam vs Meropenem for Treatment of Hospital-Acquired and Ventilator-Associated Bacterial Pneumonia (REPROVE): Analyses per US FDA–Specified End Points
Source: Open Forum Infect Dis. 2019 Apr 25;6(4):ofz149. doi: 10.1093/ofid/ofz149 (PMC6483139; doi:10.1093/ofid/ofz149)
Supplement: Supplementary-Materials [file ofz149_suppl_supplementary-materials.pdf]

Online Data Supplement

Randomized Trial of Ceftazidime-Avibactam Versus Meropenem for Treatment of Hospital-Acquired and Ventilator-Associated Bacterial Pneumonia (REPROVE): Analyses per US FDA-Specified Endpoints

Antoni Torres, MD, PhD, FERS, Doug Rank, MD, David Melnick, MD, Ludmyla Rekeda, PhD, Xiang Chen, PhD, Todd Riccobene, PhD, Ian A. Critchley, PhD, Hassan D. Lakkis, PhD, Dianna Taylor, BSc, PgC, Angela K. Talley, MD

Supplementary File 1. Statistical Procedures

Table S1. Key Inclusion and Exclusion Criteria

Table S2. CAZ-AVI Dosage Regimens for Patients With MSRIB (CrCl 16–50 mL/min) Before and After Protocol Amendment

Table S3. Secondary Outcome Variables

Table S4. Clinical and Microbiological Outcomes: Definitions

Table S5. Analysis Populations: Definitions

Table S6. Gram-Negative Aerobic Pathogens Isolated at Baseline (Micro-ITT Population)

Table S7. Sensitivity Analyses: 28-Day All-Cause Mortality (ITT Population)

Figure S1. REPROVE study design.

Figure S2. CAZ-AVI and ceftazidime MIC distributions of (A) Enterobacteriaceae and (B) *Pseudomonas aeruginosa* isolates at baseline (micro-ITT population).

Supplementary File 1. Statistical Procedures

Based on differing regulatory requirements between the US Food and Drug Administration (FDA) and the European Medicines Agency (EMA) for the statistical analyses of this study, 2 separate statistical analysis plans (SAPs) were developed by the study sponsors (Allergan and AstraZeneca) for the US and European Union, with input from the respective regulatory authorities; both SAPs were finalized before database lock. The US SAP described prespecified analyses based on endpoints and analysis populations as agreed upon with the FDA, while a separate SAP, prepared by AstraZeneca, detailed the approach to statistical analyses according to EMA requirements.

Determination of Sample Size

This study was expected to randomize at least 850 eligible patients, which ensured that there was sufficient power (≥90%) to test the primary hypothesis based on 10% noninferiority. The sample size was calculated using SAS software (SAS Institute, Cary, NC) using the Farrington and Manning method [1].

Noninferiority Analysis: 28-Day All-Cause Mortality

Statistical analyses for the primary endpoint, 28-day all-cause mortality in the intent-to-treat (ITT) population, were based on the Kaplan-Meier (KM) method (1). Noninferiority was tested using KM estimates of the cumulative survival of the 2 treatment arms up to the end of the Day 28 visit window (28–32 days from randomization). The statistical test of noninferiority for the primary efficacy analysis was performed at the 2.5% one-sided significance level. Noninferiority of ceftazidime-avibactam (CAZ-AVI) to meropenem with respect to the primary endpoint was to be concluded if the upper limit of the 2-sided 95% CI for the difference in 28-day all-cause mortality rates between the treatments (CAZ-AVI minus meropenem) was <10%. The 2-sided 95% CIs for the difference in mortality rates (based on the KM estimates) were calculated based on Greenwood's variance estimates [1]. Type I error was controlled at 5% using a sequential statistical testing procedure.

Sensitivity Analyses

To assess the impact of patients who were lost to follow-up before the Day 28 visit on the primary analysis, sensitivity analyses of the primary endpoint were performed, excluding patients lost to follow-up before the Day 28 visit, using the unstratified Miettinen and Nurminen method (2), and using multiple imputations for patients with missing data, where missing mortality status was imputed as alive at Day 28 for patients with a favorable clinical response at end of treatment (EOT) and/or test of cure (TOC) visits, and otherwise was assumed to follow a binomial distribution consistent with the observed mortality rate for the assigned treatment arm.

Parallel analyses of 28-day all-cause mortality were performed for the microbiological ITT (micro-ITT) population using similar methods as described above for the primary efficacy and sensitivity analyses in the ITT population and were utilized as supportive of the primary ITT analysis.

Noninferiority Analysis: Clinical Cure at the TOC Visit

The statistical test of noninferiority for the key secondary efficacy analysis (proportion of patients with clinical cure at the TOC visit [21–25 days from randomization] in the ITT population) was performed at the 2.5% one-sided significance level. Noninferiority was to be concluded if the lower limit of the 2-sided 95% CI for the difference in clinical cure rates between the 2 treatment arms was greater than –10%. The 2-sided 95% CIs for the observed difference in clinical cure rates were computed using the unstratified Miettinen and Nurminen method (2). Type I error was controlled at 5% using a sequential statistical testing procedure.

Subgroup Analyses

Analyses of the primary efficacy variable (28-day all-cause mortality) were performed in the ITT and micro-ITT population for key subgroups of interest by baseline patient and disease characteristics, that is, Acute Physiology and Chronic Health Evaluation (APACHE) II category, ventilation status, baseline renal function category (including moderate to severe renal impairment at baseline $[MSRIB]_{orig}$, $MSRIB_{new}$, and augmented renal clearance), infection type (hospital-acquired bacterial pneumonia [HAP] or ventilator-associated bacterial pneumonia [VAP] diagnosis), region, prior systemic antibiotic use, and receipt of concomitant aminoglycoside therapy. The impact of prior systemic Gram-negative antibiotic use in the 72 hours before randomization and concomitant aminoglycoside use up to EOT was further assessed in an exploratory post hoc analysis, with patients assigned to exposure categories by blinded review of prebaseline and postbaseline data, evaluated as to whether patients received prior and/or concomitant therapy with potential activity against their baseline pathogen. For baseline pathogens for which susceptibility data to the prior or concomitant antibiotic received were unknown or unavailable, exposure for that patient was conservatively categorized as having received potentially effective therapy.

Descriptive summaries were provided where appropriate for primary and secondary efficacy variables, with between-arm difference and 2-sided 95% CIs produced using the unstratified Miettinen and Nurminen method [2]. No statistical inferential tests were performed for analysis of safety and tolerability data.

Table S1. Key Inclusion and Exclusion Criteria

Key Inclusion Criteria	Key Exclusion Criteria
• Onset of symptoms ≥48 hours after admission or <7 days following discharge<br>• Radiograph finding of new or worsening infiltrate within 48 hours before randomization<br>• ≥1 systemic sign of infection<br>- Fever (temperature >38°C) or hypothermia (temperature <35°C)<br>- White blood cell count >10,000 or <4500 cells/mm^3, or >15% band forms<br>• ≥2 respiratory signs or symptoms<br>- New-onset cough or worsening of cough<br>- Purulent sputum or endotracheal secretions<br>- Auscultatory findings consistent with pneumonia/pulmonary consolidation (eg, rales, rhonchi, bronchial breath sounds, dullness to percussion, egophony)<br>- Dyspnea, tachypnea, or hypoxemia (O_2 saturation <90% or pO_2 <60 mm Hg while breathing room air)<br>- Need for mechanical ventilation or acute changes to ventilator support<br>• Specimen obtained from respiratory tract for Gram stain and culture within 48 hours before randomization and following onset of signs and symptoms	• Isolation of Gram-positive pathogen only, or Gram-negative pathogen not expected to respond to CAZ-AVI and/or meropenem, or known carbapenem-resistant Gram-negative pathogen<br>• Total antibiotic exposure >24 hours for antibiotics whose administration began in the 48 hours before randomization<br>• Expected treatment course >14 days<br>• APACHE II score >30 or <10<br>• High likelihood of death based on investigator clinical judgment<br>• Expected need for dialysis or creatinine clearance <16 mL/min

APACHE II=Acute Physiology and Chronic Health Evaluation II; CAZ-AVI=ceftazidime-avibactam; pO_2=partial pressure of oxygen.

Table S2. CAZ-AVI Dosage Regimens for Patients With MSRIB (CrCl 16–50 mL/min) Before and After Protocol Amendment

Renal Function Category at Baseline	Estimated CrCl, (mL/min)[a]	Original Protocol[b] ($MSRIB_{orig}$)	Amended Protocol[b] ($MSRIB_{new}$)
Moderate renal impairment	31–50	1.25 g (1 g ceftazidime and 0.25 g avibactam) IV q12h	1.25 g (1 g ceftazidime and 0.25 g avibactam) IV q8h
Severe renal impairment	16–30	1.25 g (1 g ceftazidime and 0.25 g avibactam) IV q24h	0.94 g (0.75 g ceftazidime and 0.19 g avibactam) IV q12h

CAZ-AVI=ceftazidime-avibactam; CrCl=creatinine clearance; MSRIB=moderate to severe renal impairment at baseline; qXh=every X hours.

[a]Baseline CrCl as calculated using the Cockcroft-Gault formula rounded to the nearest whole number.

[b]All CAZ-AVI doses were administered as a 2-hour infusion.

CAZ-AVI dosing for patients with CrCl >50 mL/min was consistent with the labeled recommendations [3] throughout the study. Meropenem dosing and dose adjustments for renal impairment were consistent with labeled dosage recommendations [4]; all meropenem doses were administered as a 30-minute infusion.

Table S3. Secondary Outcome Variables

Clinical Evaluation	Outcome Variable	Assessment Visits	Population(s)	Subsets
All-cause mortality	Deaths	Day 28 (FPFU)	Micro-ITT	Patients with CAZ-NS pathogens
All-cause mortality	Deaths	Day 28 (FPFU)	CE	
Clinical response	Clinical cure	EOT, TOC	ITT, micro-ITT, CE, ME	
Clinical response	Clinical cure	TOC	Micro-ITT, ME	By baseline pathogen; Patients with CAZ-NS pathogens
Per-patient microbiological response	Favorable: eradication or presumed eradication	EOT, TOC	Micro-ITT, ME	Patients with CAZ-NS pathogens
Per-pathogen microbiological response	Favorable: eradication or presumed eradication	EOT, TOC	Micro-ITT, ME	By baseline pathogen; by baseline CAZ-NS pathogen; by CAZ-AVI/meropenem MIC category
Safety, tolerability	AE, laboratory, ECG data	EOT, FPFU	Safety	

CAZ-AVI=ceftazidime-avibactam; CAZ-NS=ceftazidime-nonsusceptible; CAZ-S=ceftazidime-susceptible; CE=clinically evaluable; EOT=end of treatment; FPFU = final patient follow up; ITT=intent to treat; ME=microbiologically evaluable; MIC=minimum inhibitory concentration; micro-ITT=microbiological ITT; TOC=test of cure.
Definitions of analysis populations can be found in **Table S5**.

Table S4. Clinical and Microbiological Outcomes: Definitions

Clinical Response	EOT Visit	TOC Visit
Clinical cure	Patients were considered to have a favorable clinical response if:<br>1. The patient was alive, and all signs and symptoms of pneumonia had resolved or improved such that all antibacterial therapies for HAP/VAP were stopped, and<br>2. No antibacterial therapy other than those outlined by the protocol had been administered for HAP/VAP before EOT	Patients were considered to have a favorable clinical response if:<br>1. The patient was not a clinical failure at EOT and the patient was alive, and<br>2. All signs and symptoms of pneumonia had resolved or improved to an extent that no antibacterial therapy for HAP/VAP had been taken between the EOT and TOC visits, inclusive
Clinical failure	Patients who met any 1 of the following criteria were considered to be a treatment failure for clinical response:<br>1. Mortality due to HAP/VAP between Day 3 of study therapy and EOT visit, inclusive<br>2. Incomplete clinical resolution or worsening of HAP/VAP-specific signs and symptoms that required additional antibacterial therapy for HAP/VAP at or before EOT<br>3. Development of infectious complications of pneumonia such as empyema or lung abscess after Day 2	Patients who met any 1 of the following criteria were considered to be a treatment failure for clinical response:<br>1. Designated as a clinical failure at an earlier time point (eg, EOT)<br>2. Mortality due to HAP/VAP between Day 3 of study therapy and TOC visit, inclusive<br>3. Persistence, incomplete clinical resolution, worsening, or recrudescence of HAP/VAP-specific signs and symptoms that required initiation of antibacterial therapy for HAP/VAP between the EOT and TOC visits, inclusive<br>4. Development of complications of HAP/VAP such as empyema or lung abscess at or before the TOC visit
Indeterminate	Patients who met any 1 of the following criteria were considered to have an indeterminate clinical response:<br>1. Patient lost to follow-up or assessment was not undertaken, so that a determination of clinical response could not be made	Patients who met any 1 of the following criteria were considered to have an indeterminate clinical response:<br>1. Patient lost to follow-up on or before the TOC visit or the assessment was not undertaken, so that a determination of clinical response could not be

	2. Death on or before the date of EOT visit where HAP/VAP was clearly noncontributory<br>3. Death on Study Day 1 or Day 2<br>4. Identification of an infectious complication of pneumonia such as empyema or lung abscess between Day 1 and Day 2, inclusive	made<br>2. Death on or before the date of the TOC visit where HAP/VAP was clearly noncontributory<br>3. Death on Study Day 1 or Day 2, inclusive<br>4. Identification of an infectious complication of pneumonia such as empyema or lung abscess between Day 1 and Day 2, inclusive
**Microbiological Response Classification at EOT and TOC Visits[a]**		
Favorable	Eradication	Source specimen demonstrated absence of the original baseline pathogen
	Presumed eradication	Source specimen was not available to culture, and the patient was assessed as a clinical cure
Unfavorable	Persistence	Source specimen demonstrated continued presence of the original baseline pathogen
	Persistence with increasing MIC	Continued presence of the causative organism in a source specimen obtained during or on completion of treatment with study drug and displaying a ≥4-fold higher MIC to study drug after treatment with study drug
	Presumed persistence	Source specimen was not available to culture, and the patient was assessed as a clinical failure
Indeterminate	Indeterminate	Source specimen was not available to culture, and the patient's clinical response was assessed as indeterminate

EOT=end of treatment; HAP=hospital-acquired bacterial pneumonia; MIC=minimum inhibitory concentration; TOC=test of cure; VAP=ventilator-associated bacterial pneumonia.

[a]Per-patient (overall) microbiological response at the EOT and TOC visits was determined based on individual outcomes for each baseline pathogen from respiratory or blood source.

Table S5. Analysis Populations: Definitions

Intent-to-Treat (ITT) Population
All randomized patients who received any amount of study drug.

Microbiological ITT (micro-ITT) Population
Subset of the ITT population comprising patients with a properly obtained respiratory culture demonstrating Gram-negative pathogens. Excluded were patients not expected to respond to either study drug (ie, patients with only the following monomicrobial Gram-negative infections: any of the *Acinetobacter* species, any *Legionella* species, *Stenotrophomonas maltophilia*, *Elizabethkingia meningoseptica*). Included were patients without baseline respiratory cultures or no respiratory pathogen identified but with a Gram-negative organism known to cause pneumonia identified from baseline blood cultures.

Clinically Evaluable (CE) Population
Subsets of the ITT population at EOT and TOC visits. Patients included in the CE population at Day 28 (required for assessment of mortality) were assumed to be the same as in the CE population at the TOC visit.
Included:

- Patients with properly obtained baseline respiratory or blood cultures demonstrating Gram-negative pathogens with or without concomitant Gram-positive pathogens (excluding patients with Gram-negative pathogens not expected to respond to either study drug (as for the micro-ITT population)

AND also

- Patients from whom no etiologic pathogens were identified from respiratory or blood cultures at baseline
- Patients who received an adequate course of treatment:
  EITHER
  0≥48 hours, with ≥80% of the scheduled drug administered over the number of days administered
  OR
  0<48 hours before discontinuing treatment because of an AE
- Had a clinical response of cure or failure (ie, excluding indeterminate) within the per-protocol visit window for the respective EOT and TOC visits (ie, clinical response assessment within 24 hours of the last dose for EOT or from Days 21–25 for TOC)
- Had no protocol deviations that would affect assessment of efficacy
- Did not receive prior antibiotics other than as outlined as acceptable by the CSP
- Did not receive concomitant antibiotic therapy with potential activity against the baseline pathogen, except for protocol-allowed antibiotics; if no baseline pathogen was identified, receipt of any antibiotic other than those outlined in the CSP excluded the patient from the CE populations.

**Microbiologically Evaluable (ME) Population**<br>Subsets of the CE population defined separately for each of the EOT and TOC visits included patients who had at least one etiologic pathogen from an adequate baseline culture that was susceptible to both ceftazidime-avibactam and meropenem.
**Safety Population**<br>All randomized patients who received any amount of study drug and were analyzed according to treatment received.

AE=adverse event; CE=clinically evaluable; CSP=clinical study protocol; EOT=end of treatment; ITT=intent to treat; ME=microbiologically evaluable; micro-ITT=microbiological ITT; TOC=test of cure.

Table S6. Gram-Negative Aerobic Pathogens Isolated at Baseline (Micro-ITT Population)

**Pathogen group/pathogen[a,b]**	**CAZ-AVI (N=187) n (%)**	**Meropenem (N=195) n (%)**
Enterobacteriaceae	133 (71.1)	147 (75.4)
Enterobacter aerogenes	8 (4.3)	9 (4.6)
Enterobacter cloacae	29 (15.5)	23 (11.8)
Escherichia coli	22 (11.8)	23 (11.8)
Klebsiella pneumoniae	65 (34.8)	75 (38.5)
Proteus mirabilis	14 (7.5)	12 (6.2)
Serratia marcescens	15 (8.0)	13 (6.7)
Gram-negative pathogens other than Enterobacteriaceae	85 (45.5)	84 (43.1)
Haemophilus influenzae	16 (8.6)	25 (12.8)
Pseudomonas aeruginosa	64 (34.2)	51 (26.2)
CAZ-NS pathogens[c]	49 (26.2)	59 (30.2)
Enterobacteriaceae	36 (19.3)	45 (23.1)
E. aerogenes	4 (2.1)	2 (1.0)
E. cloacae	6 (3.2)	6 (3.1)
E. coli	6 (3.2)	8 (4.1)
K. pneumoniae	22 (11.8)	31 (15.9)
P. aeruginosa	12 (6.4)	16 (8.2)

CAZ-AVI=ceftazidime-avibactam; CAZ-NS=ceftazidime-nonsusceptible; CLSI=Clinical and Laboratory Standards Institute; micro-ITT=microbiological intent to treat.

[a]Respiratory tract or blood source. Only pathogens isolated from ≥10 patients across treatment arms (≥5 for CAZ-NS subset) are presented. [b]Patients may have had >1 pathogen isolated. Multiple isolates of the same species from the same patient are counted only once, regardless of source (respiratory tract or blood).[c]CAZ-NS designation determined according to CLSI criteria for ceftazidime-resistant and -intermediate categories [5].

Table S7. Sensitivity Analyses: 28-Day All-Cause Mortality (ITT Population)

Response	Deaths, n (%) CAZ-AVI (n=425)	Deaths, n (%) Meropenem (n=428)	Difference (%) (95% CI)
Number of patients who died (all-cause mortality), excluding patients lost to follow-up before the Day 28 visit	42 (9.9)	36 (8.4)	1.5 (–2.4, 5.4)[a]
Number of patients who died (all-cause mortality), using multiple imputation of missing data at the Day 28 visit	43 (9.9)	36 (8.3)	1.5 (–2.3, 5.4)[b]

CAZ-AVI=ceftazidime-avibactam; ITT=intent to treat.

[a]Based on difference between treatment arms in proportions of patients who died up to Day 28; CIs for the difference were calculated using the unstratified Miettinen and Nurminen method.

[b]CIs were calculated using the standard multiple imputation method (Supplementary File 1).

Figure S1. REPROVE study design.

CAZ-AVI=ceftazidime-avibactam; EOT=end of treatment; FDA=US Food and Drug Administration; FPFU=final patient follow-up; HAP=hospital-acquired bacterial pneumonia; ITT=intent to treat; IV=intravenous; NI=noninferiority; q8h=every 8 hours; TOC=test of cure; VAP=ventilator-associated bacterial pneumonia.

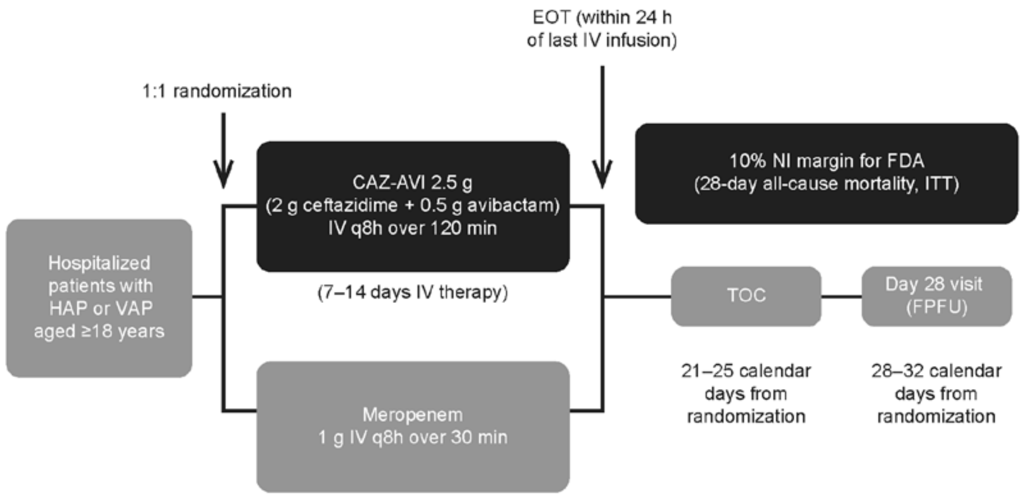

Figure S2. CAZ-AVI and ceftazidime MIC distributions of (A) Enterobacteriaceae and (B) *Pseudomonas aeruginosa* isolates at baseline (micro-ITT population).

CAZ-AVI=ceftazidime-avibactam; MIC=minimum inhibitory concentration; micro-ITT=microbiological intent to treat.

A

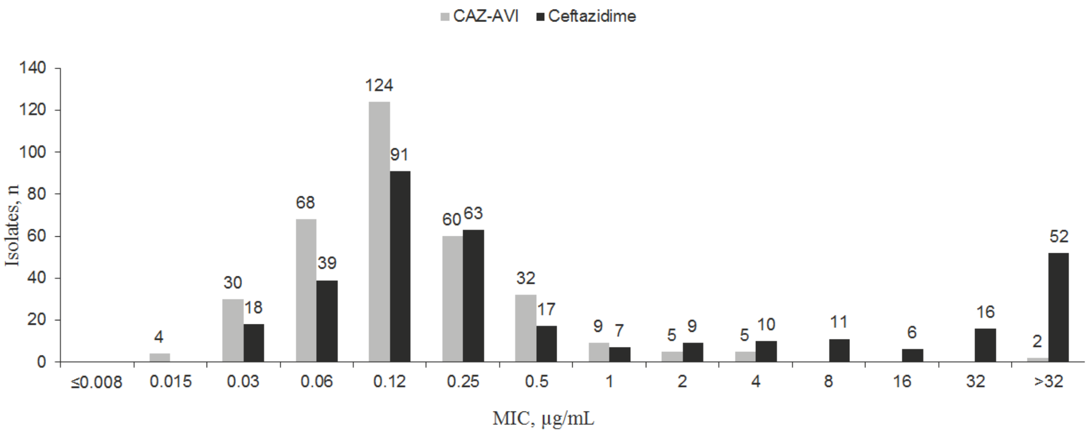

B

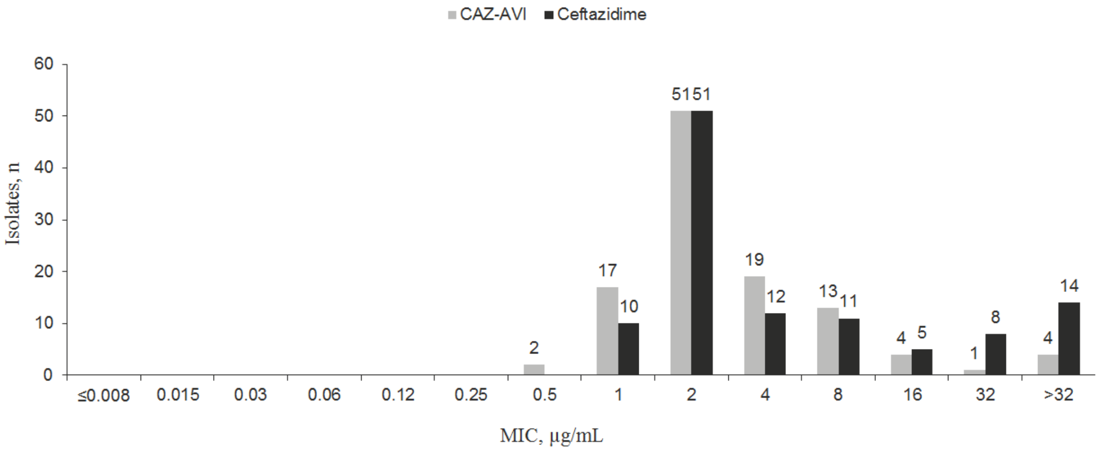

References

1. Flawless J. Statistical models and methods for lifetime data. New York: John Wiley & Sons; **1982**.

2. Miettinen O, Nurminen M. Comparative analysis of two rates. *Stat Med*. **1985**; 4: 213-226.

3. AVYCAZ (ceftazidime-avibactam). Full Prescribing Information, Allergan plc, Dublin, Ireland, 2018.

4. Merrem IV (meropenem for injection). Full Prescribing Information, AstraZeneca, Wilmington, DE, 2016.

5. Clinical and Laboratory Standards Institute. Performance standards for antimicrobial susceptibility testing; Twenty-seventh Informational Supplement. CLSI document M100-S27: Clinical and Laboratory Standards Institute; 2017.
